# 535. Risk Factors for Seeking Medical Care Following Monoclonal Antibody Treatment For COVID-19

**DOI:** 10.1093/ofid/ofad500.604

**Published:** 2023-11-27

**Authors:** I S M A I L O MALIK, Susan M Szpunar, Natali Salaytah, Ashish Bhargava, Mamta Sharma

**Affiliations:** ASCENSION ST. JOHN HOSPITAL, GROSSE POINTE WOODS, Michigan; Ascension St. John Hospital, MI; ascension st john, gross pointe woods, Michigan; Ascension St. John Hospital; Wayne State University School of Medicine, Grosse Pointe Woods, Michigan; Ascension St John Hospital & Medical Center, Grosse Pointe Woods, MI. 48236, Michigan

## Abstract

**Background:**

The FDA issued emergency use authorization for neutralizing monoclonal antibodies (mAb) for the treatment of mild-moderate coronavirus disease 2019 (COVID-19) in patients who were at high risk of disease progression.

**Methods:**

In this case-control study, we assessed risk factors for patients with mild-moderate COVID-19 infection treated with mAb on the composite outcome of subsequent evaluation in the Emergency Department (ED) or inpatient admission through December 1, 2020, through May 30, 2022. Cases were patients who failed MAB and visited the ED or were hospitalized within four weeks of their MAB treatment; controls received MAB but did not visit the ED or were not hospitalized within four weeks of MAB treatment. Cases were matched to controls in 1:1 ratio for the patient age ± 5 years. and for the calendar month of positive COVID tests. Data were analyzed using Student’s t-test, the chi-squared test, the Mann-Whitney U test and logistic regression using SPSS v. 29.0. The study was approved by the Ascension IRB.

**Results:**

Of 212 patients who received mAb treatment, the mean (SD) age was 61.3 (±18) years; 51.8 % (99) were female, and 84.8% (162) white. The mean body mass index of the cohort was 32.6 ± 8.5 kg/m2. Following mAb infusion, 106 cases had a positive composite outcome of interest. Patients with peptic ulcer disease (PUD) (64.4% vs 35.6%; p=0.029), asthma (72.7% vs 27.3%; p=0.024), and chronic lung diseases (12.5% vs 35.5%; p=0.001) were more likely to seek medical care in the ED or hospital post mAb infusion. Alcohol users were less likely to seek medical care following mAb infusion (41.7% vs 58.3%; p=0.013). The number of reported days of symptoms prior to mAb infusion were 5.81 days in cases versus 5.79 days in controls (p=0.98). There were no differences in sex, BMI, tobacco use or vaccinations rates between the two groups. In multivariable logistic regression, factors associated with seeking medical care post mAb infusion were Black race (OR 3.1; 95% CI 1.1-9.2), peptic ulcer disease (OR 3.3; 95% CI 1.1-9.7) and alcohol use at the time of mAb treatments (OR 0.5; 95% CI 0.3-0.97)

**Conclusion:**

Independent risk factors for seeking medical care following mAb treatment for COVID-19 were Black race and peptic ulcer disease while alcohol users were less likely to seek medical care following the mAb infusion.

**Disclosures:**

**All Authors**: No reported disclosures

